# A Survey Among Italian Physicians During COVID-19 Outbreak. Could Bacillus Calmette–Guérin Vaccine Be Effective Against SARS-CoV2?

**DOI:** 10.3389/fphar.2021.646570

**Published:** 2021-05-03

**Authors:** Vincenzo Patella, Alessandro Sanduzzi, Dario Bruzzese, Giovanni Florio, Raffaele Brancaccio, Gabriella Fabbrocini, Gabriele Delfino

**Affiliations:** ^1^ Division of Allergy and Clinical Immunology, Department of Medicine ASL Salerno, “Santa Maria Della Speranza” Hospital, Salerno, Italy; ^2^ Postgraduate Program in Allergy and Clinical Immunology, University of Naples Federico II, Naples, Italy; ^3^ Department of Clinical Medicine and Surgery, Section of Respiratory Disease, University of Naples Federico II, Naples, Italy; ^4^ Staff of UNESCO Chair on Health Education and Sustainable Development, University Federico II, Naples, Italy; ^5^ Department of Public Health, University of Naples Federico II, Naples, Italy; ^6^ Department of Clinical Medicine and Surgery, Dermatology, Section of Dermatology, University of Naples Federico II, Naples, Italy; ^7^ Laboratory of Clinical Biochemistry, Monaldi Hospital, Naples, Italy

**Keywords:** SARS-CoV2, COVID-19, trained immunity, natural killer cells, innate immunity, epidemiology, Bacille Calmette-Guerin vaccine

## Abstract

**Background:** Epidemiological studies show that BCG-vaccinated population seems to be more likely protected from COVID-19 infection, but WHO gave a stark warning on use of BCG vaccine without confirmed COVID-19 trials. The aim of the study is to evaluate whether TB vaccination, performed several years earlier, could confer protection against COVID-19.

**Methods:** After the Ethical Committee authorization, professional orders were used to contact physicians with an online survey. Specialty, COVID-19 infection and previous BCG vaccination were recorded. Statistical data analysis was performed.

**Results:** 1906 physicians answered the questionnaire, (M = 1068; F = 838; mean age 50.7 ± 13.3 years; range 24–87), more than half (1062; 55.7%) experienced BCG vaccination. Professional activity was recorded, and only 49 subjects (2.6%) of them were infected by SARS-CoV2. Among the group of infected people, asymptomatic form occurred in 12 subjects (24.5%); a pauci-symptomatic form in 24 subjects (49.0%); and a severe form (pneumonia and/or respiratory distress) in 13 (26.5%). Considering only the clinically relevant form of COVID-19, period prevalence was 2.2% (23/1062) in the vaccinated group and 1.7% (14/844) in the unvaccinated group (OR: 1.31, 95% C.I.: 0.68–2.63, *p* = 0.427).

**Conclusion:** Our experience does not confirm the possible protective role of BCG vaccination, performed years earlier, against COVID-19. Although recent epidemiological studies point out in BCG-vaccinated population a lower prevalence of SARS-CoV2 infection, in our cohort of physicians no significant difference was found in terms of prevalence of COVID-19 infection. Our data underline the necessity to follow the WHO warning about the indiscriminate use of BCG vaccine, until clear evidence of protection by BCG vaccination against COVID-19 is fully demonstrated.

## Introduction

At the end of December 2019, the Chinese authorities informed the World Health Organization (WHO) of a series of pneumonia-like cases in the city of Wuhan, likely originating from a fish and animal market in the city (Li et al., 2020). Only in January 2020, the first news about the viral outbreak detected in the city of Wuhan was confirmed by the Chinese government, about a new virus belonging to the coronavirus family (Coronaviridae Study Group of the International Committee on Taxonomy of Viruses, 2020). Subsequently, the infection spread rapidly throughout the world with a significant intensity of infection in Europe, especially in Italy (Grasselli et al., 2020). On March 11, 2020, the General Director of the WHO Tedros Adhanom Ghebreyesus, given the dramatic increase in the cases and countries involved, declared COVID-19 a pandemic (WHO Director-General’s opening remarks at the media briefing on COVID-19”, 2020).

An old vaccine, *Bacillus* Calmette–Guérin (BCG), a live attenuated strain of *Mycobacterium bovis*, was originally developed by Albert Calmette and Camille Guérin at the beginning of the 20th century, with the aim to prevent tuberculosis (TB) (Calmette et al., 1927). It is well known that BCG vaccine protects against serious tuberculosis disease for up to 15 years after vaccination, while in some cases is more effective than it was thought, offering protection for at least 20 years (Mangtani et al., 2018).

In recent weeks, some studies hypothesize whether BCG vaccine can protect against SARS-CoV2 infection. There are a lot of expert opinions, but available data related to epidemiological studies are limited and controversial (Faust et al., 2020; Hamiel et al., 2020; Ozdemir et al., 2020). Several trials are currently investigating the hypothesis that heterologous immune response induced by BCG could protect against severe COVID-19.^1^ ^,^ ^2^

In addition to inducing a specific immune response against Mycobacterium tuberculosis, anti-TB vaccination with BCG also seems to promote a nonspecific protection against other viral and bacterial agents (Biering-Sørensen et al., 2012; Hirve et al., 2012), acting as an immune enhancer, in particular on the innate component of the immune system. This phenomenon, called “trained immunity,” has been studied extensively from an immunological point of view. The induced “non–antigen-specific immunological memory” in innate immunity cells such as macrophages, monocytes, and natural killer cells can be the effector of this phenomenon (Ponticiello et al., 2014; Netea et al., 2016). After stimulation with BCG these cells undergo an epigenetic reprogramming of some transcription factors; promoters of cytokine genes are de-phased or *de novo* created. This epigenetic upgrade is maintained even after the disappearance of the primary stimulus (“non–antigen-specific innate immune memory”), giving to these cells the ability to respond more powerfully to a secondary stimulus, even if not correlated with the primary stimulus (Uthayakumar et al., 2018; Netea et al., 2020). All these mechanisms could justify a protective potential of “trained immunity” against SARS-CoV2.

In this study, we investigated with a questionnaire the relationship between BCG vaccine and SARS-CoV2 infection among a group of Italian physicians, who still currently are highly exposed to SARS-CoV2 (Patella et al., 2020c). In Italy, anti-TB vaccination is not mandatory, but it was up to few years ago, only for students of Medicine and Dentistry who were starting their degree course; for this reason most physicians have been vaccinated with BCG.

The aim of the study is to evaluate whether TB vaccination, performed several years earlier, could confer protection against COVID-19. The results could confirm recent epidemiological observations according to which in countries where the vaccine is performed in childhood (therefore many years before), there is a lower spread of COVID-19 (Gursel and Gursel, 2020; Ozdemir et al., 2020).

## Materials and Methods

### Ethics

The studies for this survey involving human participants were reviewed and approved by an Independent Ethics Committee: “*Comitato Etico Interaziendale Campania SUD*” (Note: n. 19_53, 2020).

### Questionnaire

The anonymous questionnaire was administered to all the medical doctors belonging to the Campania region (Southern Italy) Professional Orders (44,203 subjects, at time of survey), reached through their mailing list, by a computer-based platform with a survey technique. Participation was voluntary and uncompensated; after giving informed consent, the participants could access the questionnaire. To avoid incomplete questionnaires, the registration of the questionnaire was possible only if all questions were answered.

A web-based data collection tool was used to collect non-identified data over the period between April 10 and May 7, 2020. Data collected included each individual subject’s gender, age, type of care activity carried out, results of nasopharyngeal swab, and serological examination, any contagion with SARS-CoV2 (considered in case of positive swab or serology), the level of the clinical signs and symptoms in case of infection, the previous BCG vaccine, and the year of vaccination (Table 1).

**TABLE 1:** Questionnaire.

1. Basic epidemiological data
Age, years
Sex
Type of care activity
A. Intensive care or first aid doctor
B. Hospital internal medicine physicians
C. Dentistry
D. Doctor working with discharged patients during the pandemic
E. General practitioner and pediatrician
F. Outpatient specialist
G. Other
2. Did you receive the swab for COVID-19?
3. If yes, indicate the result among these:
Positive
Negative
4. Did you receive the serological examination for COVID-19?
5. If yes, indicate the result among these:
Negative
Positive only for IgM
Positive only for IgG
Positive for both IgM and IgG
6. Have you been infected by the new SARS-CoV2? (answer yes only in case of positive swab and/or serology)
7. If yes, indicate the clinical feature among the following:
Asymptomatic form
Pauci-symptomatic form (fever> 37.5°, cough, and cooling symptoms)
Pneumonia and/or respiratory distress
8. Did you receive BCG vaccine?
9. If yes, indicate the year of execution of the vaccine (if you do not remember the year, indicate the year of enrolment in the degree course)

### Statistical Analysis

Statistical platform R (vers. 3.5.1) was used for all the statistical analyses. Descriptive statistics were used to characterize the sample; mean ± standard deviation or median [25th; 75th percentile] with range in case of numerical variables and absolute frequencies with percentages in case of categorical factors. Association between vaccination and infection was assessed using the chi-square test and further quantified using odds ratio (OR) with the corresponding 95% confidence intervals (95% CIs). Logistic regression models were used to adjust the association between vaccination and infection for potential confounding factors (age, gender, and care activity). Statistical significance was set at *p* < 0.05.

## Results

### Epidemiological Data and Bacille Calmette–Guerin Vaccination

The questionnaire collection phase lasted one month, from April 10 to May 7, 2020. During this period, a total of 1906 physicians joined the study, corresponding to a participation rate in the survey of 4.3%: 1068 men (56.0%) and 838 (44.0%) women. The mean age (±standard deviation) was 50.7 ± 13.3 years, with a minimum age of 24 years and a maximum age of 87 years. In the whole sample, more than half (55.7%) had undergone BCG vaccine. Median age at vaccination was 19 years [18; 20] (Table 2), which is in line with the age at which medical students enroll in the degree course. Vaccination coverage, stratified by gender and age groups, is reported in Table 3. The highest coverage was observed in the middle-age class (41–60 years) and a slightly higher prevalence of BCG vaccination was observed in the female sample.

**TABLE 2:** Population Characteristic.

Gender	
Male	838 (44%)
Female	1068 (56%)
Age, years	50.7 ±13.3 (24–87)
Age class	
≤40	528 (27.7%)
41–60	783 (41.1%)
>60	595 (31.2%)
Care activity	
Intensive care or first aid doctor	136 (7.1%)
Hospital internal medicine physicians	251 (13.2%)
Dentistry	283 (14.9%)
Doctor working with discharged patients during the pandemic	64 (3.4%)
General practitioner and pediatrician	332 (17.4%)
Outpatient specialist	192 (10.1%)
Other	648 (34%)
Year of vaccination	
Overall	1062 (55.7%)
After 2000	145 (7.6%)
After 2005	54 (2.8%)
Age at vaccination	19 years [18; 20]
Time since vaccination	34 years [24; 43]
Number of swabs	385 (20.2%)
Number of serological tests	453 (23.8%)

**TABLE 3:** Vaccination coverage in the sample stratified by gender and age groups.

Age class	Overall (%)	Male (%)	Female (%)
≤30	22 (14)	8 (12.9)	14 (14.7)
31–40	196 (52.8)	63 (47.4)	133 (55.9)
41–50	264 (71.7)	120 (71.4)	144 (72)
51–60	253 (61)	155 (58.7)	98 (64.9)
60–70	302 (55.6)	213 (54.2)	89 (59.3)
>70	25 (48.1)	23 (47.9)	2 (50)
Overall	1062 (55.7)	582 (54.5)	480 (57.3)

In Italy, the mandatory TB vaccination became voluntary for medical students in 2001. Students who enroll in medicine are usually about 19 years old, and BCG vaccination was performed at that exact moment. Considering the end of the obligation in 2001, all the students enrolled at that time and who were no longer required to take the vaccine would now be around 40 years old. Therefore, analyzing the sample by age, we noticed that, in the group of subjects <40 years, the number of vaccinated people is significantly lower (194/491—39.5%) than the group ≥40 (866/1415—61.2%).

### Infected Population and Period Prevalence with Respect to Care Activity

Forty-nine physicians were infected with SARS-CoV2 out of the sample analyzed of 1906 subjects, with a period prevalence of 2.6% (Table 4), a much higher value than in the general population of the Campania region (0.08%) calculated on the basis of the cumulative data of the Civil Protection Department on May 07, 2020.

**TABLE 4:** Infection rates related to gender, age, and care activity.

	Infection rate	
	All forms of infection	Clinically relevant infections
Overall	49 (2.6)	37 (1.9)
Gender		
Male	25 (2.3)	20 (1.9)
Female	24 (2.9)	17 (2)
Age class		
≤40	19 (3.6)	13 (2.5)
41–60	21 (2.7)	17 (2.2)
>60	9 (1.5)	7 (1.2)
Care activity		
Intensive care or first aid doctor	7 (5.1)	6 (4.4)
Hospital internal medicine physicians	15 (6)	12 (4.8)
Dentistry	4 (1.4)	4 (1.4)
Doctor working with discharged patients during the pandemic	3 (4.7)	2 (3.1)
General practitioner and pediatrician	4 (1.2)	2 (0.6)
Outpatient specialist	3 (1.6)	2 (1)
Other	13 (2)	9 (1.4)

**FIGURE 1:**
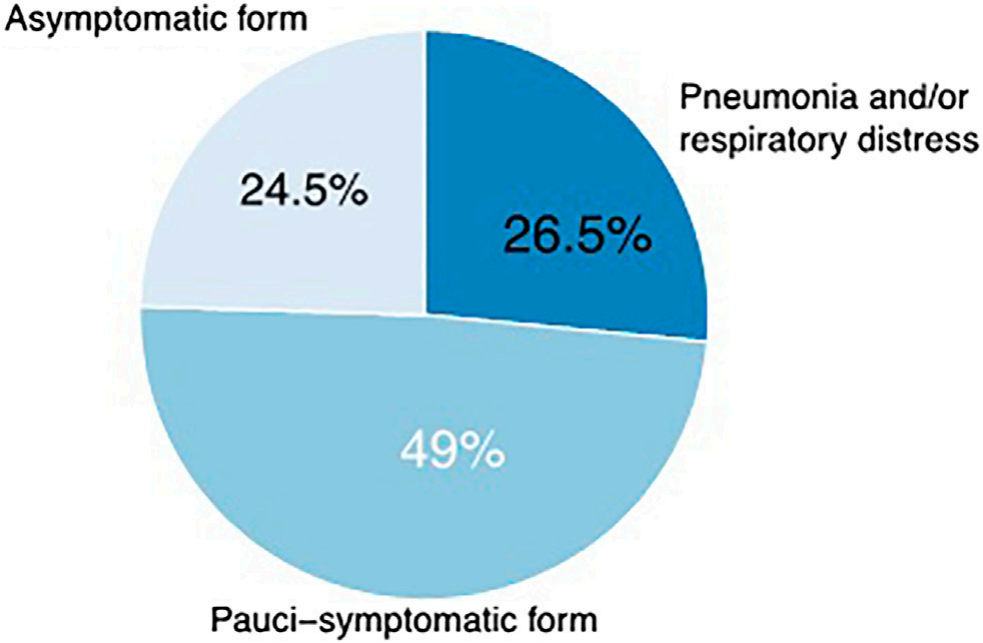
Clinical features among infected physicians. An asymptomatic state occurred in 12 subjects (24.5%); a pauci-symptomatic state in 24 subjects (49.0%); and a severe state in 13 subjects (26.5%).

The clinical features registered among infected physicians were as follows: an asymptomatic condition in 12 subjects (24.5%); a pauci-symptomatic condition characterized mainly by fever, cough, and nonspecific symptoms, which occurred in 24 subjects (49.0%); and a severe condition characterized by pneumonia and respiratory distress syndrome, which occurred in 13 subjects (26.5%) (Figure 1).

With respect to all forms of infection (Table 4), average age of infected physicians was 47.1 ± 11.4 years with the highest period prevalence (3.6%) observed in the youngest age group (≤40). Depending on the type of care activity, a different percentage of contagion with SARS-CoV2 was observed. The most at-risk categories were the physicians of the critical area, the hospital internal medicine physicians, and those working with discharged patients during the emergency, with a period prevalence of 5.1; 6.0; 4.7%, respectively. A similar pattern was observed when only clinically relevant infections were considered (Table 4).

### Relationship Between Bacille Calmette–Guerin Vaccine and Clinically Relevant COVID-19

In these comparisons between *vaccinated* and *unvaccinated*, we took into consideration only the cases that had any clinical relevance. In fact, subjects positive for the swab and therefore infected, but without clinical features, can be considered, in terms of hypothetical vaccine protection, in the same way as noninfected subjects.

In the “*unvaccinated*” group, 14 out of 844 subjects (1.7%) were infected by a clinical form of COVID-19 (pauci-symptomatic or respiratory distress); the rate raised to 2.2% (23 out of 1062) in the “*vaccinated*” group (unadjusted OR: 1.31, 95% C.I. 0.68–2.63; *p* = 0.427). When adjusting the analysis by age, gender, and care activity the difference remains not significant (OR: 1.35, 95% C.I.: 0.68–2.71, *p* = 0.393; Table 5).

**TABLE 5:** Impact of COVID-19 related to timing BCG vaccination^a^.

	Model 1		Model 2		Model 3	
	OR (95%C.I.)	*p*	OR (95%C.I.)	*p*	OR (95%C.I.)	*p*
Vaccination	1.35 (0.68–2.71)	0.393	2.02 (0.72–5.68)	0.184	0.74 (0.1–5.78)	0.778
Age, years						
≤40	Ref	—	Ref	—		
41–60	0.83 (0.39–1.8)	0.643	1.11 (0.48–2.57)	0.808	0.88 (0.41–1.88)	0.743
>60	0.55 (0.2–1.45)	0.225	0.71 (0.25–2.03)	0.524	0.56 (0.21–1.5)	0.247
Male gender	1.19 (0.6–2.39)	0.619	1.17 (0.58–2.33)	0.661	1.18 (0.59–2.35)	0.648
Care activity						
Intensive care or first aid doctor	Ref	—	Ref	—		
Hospital internal medicine physicians	1.15 (0.42–3.17)	0.781	1.18 (0.43–3.26)	0.746	1.11 (0.4–3.03)	0.844
Dentistry	0.31 (0.08–1.12)	0.074	0.32 (0.09–1.16)	0.082	0.3 (0.08–1.11)	0.072
Doctor working with discharged patients during the pandemic	0.72 (0.14–3.67)	0.689	0.76 (0.15–3.89)	0.740	0.74 (0.14–3.77)	0.715
General practitioner and pediatrician	0.16 (0.03–0.79)	0.025	0.16 (0.03–0.83)	0.029	0.15 (0.03–0.76)	0.022
Outpatient specialist	0.24 (0.05–1.21)	0.083	0.24 (0.05–1.22)	0.085	0.23 (0.05–1.18)	0.079
Other	0.33 (0.12–0.97)	0.043	0.35 (0.12–1.02)	0.054	0.33 (0.11–0.94)	0.038

a Model 1 considers in the vaccinated group all physicians who received BCG vaccination regardless year of vaccination.

Model 2 considers as vaccinated only those physicians who received BCG vaccination after 2000.

Model 3 considers as vaccinated only those physicians who received BCG vaccination after 2005.

OR with the corresponding 95% C.I. was estimated using logistic regression models and was adjusted for all the predictors reported in the table.

When considering in the “*vaccinated* group” only those physicians who received vaccine within the last 20 years (vaccination year after 2000), a similar pattern was observed (in this model three questionnaires were excluded because they did not report the year). In the group of “*unvaccinated*” (not vaccinated or vaccinated before 2000), 31 out of 1758 individuals (1.8%) showed a clinical form of COVID-19, and in the group of “*vaccinated,*” 6 out of 145 individuals (4.1%) showed a clinical form of COVID-19 (unadjusted OR: 2.40, 95% C.I.: 0.89–5.47; *p* = 0.054). In the full logistic model, the odds of infection were two-fold higher in the vaccinated group than in the unvaccinated group without reaching statistical significance (OR: 2.02, 95% C.I.: 0.72–5.68; *p* = 0.184; Table 5).

**FIGURE 2:**
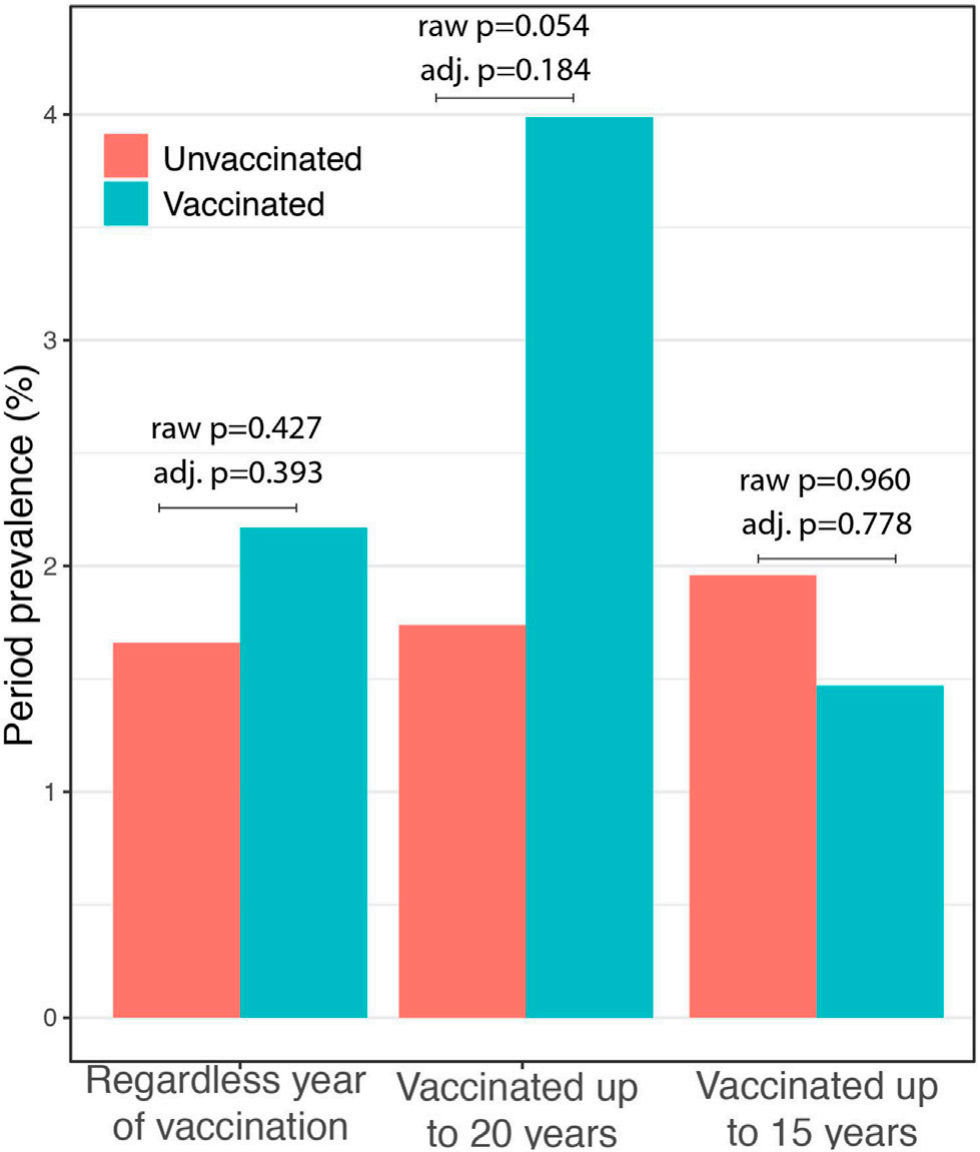
Impact of COVID-19 in relation to timing of BCG vaccination. In all three analyses, no significant difference was observed between the vaccinated and the unvaccinated group neither in unadjusted (raw *p*) or in adjusted analysis (adj. *p*).

Finally, in considering in the “*vaccinated* group,” only those physicians who received vaccine within the last 15 years (vaccination year after 2005), in the group of “*unvaccinated,*” 36 subjects out of 1849 (1.9%) showed a clinical form of COVID-19, while in the “*vaccinated*” *group*, only 1 subject out of 54 physicians (1.9%) was infected with SARS-CoV2 presenting a pauci-symptomatic form (unadjusted OR: 0.95, 95% C.I.: 0.05–4.53, *p* = 0.96). Also, in this model, three questionnaires were excluded because they did not report the year. When adjusting the analysis by age, gender, and care activity, the association remains not significant (OR: 0.74, 95% C.I.: 0.1–5.78, *p* = 0.778; Table 5; Figure 2).

### Relationship Between BCG Vaccine and Clinically Relevant COVID-19 Among the Subjects Who Had Subjected to the Swab

We also evaluated the association between BCG and COVID-19 among the subjects who had underwent the swab testing (385 subjects—20.2% of the sample): the “*vaccinated*” were 229 (and the *unvaccinated* 156). The subjects infected with SARS-CoV2 were 45 (four subjects are missing compared to the total of 49 infected because these four were judged affected only on the basis of serology), of these 34 presented a clinical form of COVID-19 (pneumonia, respiratory distress, and pauci-symptomatic form) and 11 were asymptomatic. Among “*vaccinated,*” 22 individuals (9.6%) were infected by a clinical form of COVID-19, while among “*unvaccinated,*” 12 individuals (7.7%) were infected by a clinical form of COVID-19 (OR: 1.28, 95% C.I.: 0.62–2.74; *p* = 0.516); the association remained not significant after adjusting the analysis by age, gender, and care activity (OR: 1.20, 95% C.I. 0.56–2.66; *p* = 0.6418). Taking into account year of vaccination (after 2000 or 2005) the association remained not significant both in the unadjusted and in the adjusted analysis (data not shown).

## Discussion

The results allow a rapid epidemiological review of the relationship between BCG vaccination and COVID-19 in a population exposed to SARS-CoV2. We verified that there was no difference between the vaccinated and unvaccinated population about the prevalence of the disease during the observation, which was the period of greatest exposure to such disease. In a recent study concerning general population, these epidemiological data seem to be confirmed (Hamiel et al., 2020).

In our sample, the majority of medical cohort was BCG vaccinated, and the prevalence of COVID-19 was much higher than the prevalence in regional population in the same period, confirming the high exposure of medical personnel during the COVID-19 pandemic. According to the various physicians’ categories, the most exposed to the contagion were those working in critical care (intensive care and emergency department) and internal medicine hospital. These data confirm the absolute need for protective tools in the most exposed categories (Houghton et al., 2020; Patella et al., 2020b) and the need for weighted choices in diagnostics and preventive medicine to control the infection spread among health care workers.

It should be noted that the incidence of COVID-19 differs between the various age groups regardless of the anti-TB vaccination (Table 4—“age class” section), resulting double in subjects ≤40 years of age compared to subjects >60 years. This finding could be explained by the higher exposure to the virus of younger doctors. In fact, among the subjects ≤40 years of age, 24.2% were intensive care or doctors in internist branches compared to subjects >60 where only 13.1% were at high exposure (intensive care doctor and/or internist). Moreover, among the 13 young subjects with clinically relevant forms of COVID-19, only two presented a more severe form with pneumonia and/or respiratory distress (15.4%), while among the older subjects, 4 out of 7 infected individuals (57, 1%) presented a more severe form with pneumonia and/or respiratory distress. On the other hand, the association between age class and incidence of infection is not statistically significant both in the case in which all forms of infections are considered (*p* = 0.085) and in the case of clinically relevant infections (*p* = 0.247). Moreover, considering the individual age groups, the difference in the incidence of COVID-19 between the vaccinated and unvaccinated remains insignificant.

We conducted a reassessment of the data according to three approaches related to the time elapsed since BCG vaccination. The first analysis was considered regardless of the year of vaccination; in the second analysis, we considered protection from BCG-induced “trained immunity” only who had undergone the vaccination in the last 20 years, and in the third analysis, we considered only the vaccination in the last 15 years. In all the approaches, we adjusted the analysis by age, gender, and care activity. None of the approaches showed a significant difference between the two groups.

Clearly, the study refers to vaccinations performed several years before exposure to SARS-CoV2 (on average 34 years before and in the two models, up to 20 years and up to 15 years). The immunological processes underlying “trained immunity” have a duration that is still unclear today; work by Kleinnijenhuis and Netea show that the “trained immunity status” is maintained for at least 1 year (Kleinnijenhuis et al., 2014) (the maximum time point measured), even if the duration of BCG induced “trained immunity” in terms of longevity of induced innate memory is not known. Therefore, these data can only conclude on the absence of long-term effects of BCG on COVID-19. Only trials currently underway will be able to answer the short-term effects of BCG on COVID-19.

Our study arises in response to several epidemiological observations according to which the BCG vaccine performed in childhood (therefore many years before) could protect against COVID-19, in particular it is reported that in the countries where the vaccine is regularly performed, there is a lower spread of COVID-19 (Gursel and Gursel, 2020; Ozdemir et al., 2020). Several reasons may explain the different global prevalence of the disease: for example, the difference in infections between the northern and southern hemisphere could be due, at least in part, to their different temperature, since the outbreak of COVID-19 occurred during winter in the most affected countries (Caminati et al., 2020; Wu et al., 2020). Furthermore, the main problem in studies comparing mortality rates between different countries is the different ability of national systems to report epidemiological data. In those countries with more precarious welfare systems and greater population density, where COVID-19 epidemic presented a multilevel emergency (health, social, and political), not all deaths were adequately assessed for the cause, resulting in an underestimation of COVID-19 mortality rate. This phenomenon is likely to be more frequent in poorer countries, where tuberculosis is still widespread and therefore with active BCG vaccination programs (Patella et al., 2020a).

In contrast, our study verified that a BCG vaccination performed years earlier cannot confer protection. Our data appear to be in line with the epidemiological study by Hamiel et al. conducted on a cohort of Israeli adults aged 35–41 years, who had received BCG vaccination in childhood, where the authors found a similar rate of positive test results for SARS-CoV2 compared with no vaccination (Hamiel et al., 2020). Our work suffers from the limitations of the retrospective nature of the study, and being the participation on a voluntary basis, the sample studied is not standardized. Moreover, the questionnaire-based survey, which relies only on the memory of the voluntary participant, may have introduced recall bias. Our results suffer from relatively low number of examined subjects; in particular among who had subjected to the swab, furthermore they present the limitation of not considering participants’ co-morbidities.

The possibility that BCG vaccination may protect against SARS-CoV2 infection remains an open discussion with conflicting data and opinions in the international scene (Curtis et al., 2020). Protection of tuberculosis vaccination could derive from trained immunity, a phenomenon relating to innate immunity, which, once stimulated by BCG, would drive a reprogramming of nonspecific immunological response towards even other infections, as SARS-CoV2. Epigenetic reprogramming of innate immune cells by a primary stimulus such as BCG vaccine may allow activation of transcription factors in myeloid cells (Freyne et al., 2018). In BCG vaccinated subjects, monocytes showed increased expression of activation surface markers and produced more IL-1β, IL-6, IFN-γ, and TNF-α in response to Staphylococcus aureus or *Candida* albicans (Kleinnijenhuis et al., 2012; Kleinnijenhuis et al., 2014). Kleinnijenhuis J et al. reported that NK cells isolated from volunteers 3 months after BCG vaccination, produced more pro-inflammatory cytokines on stimulation, in particular IL1β, but also IL-6 and TNFα. In another study, Kleinnijenhuis and Smith et al. reported that BCG vaccinated children showed a significant increase in surface expression of the CD69 activation marker on NK cells in response to stimulation of Pam3Cys (Smith et al., 2017).

Taken together, these data could justify the protective potential of trained immunity against SARS-CoV2. In particular, it would seem that one of the fundamental elements in the immune response against SARS-CoV2 could be just the rapidity of the pathogen elimination: indeed the escape of the immune system and the viral permanence could entail an uncontrolled response with vicarious production of inflammatory cytokines, and the clinical progression towards the most severe forms of pneumonia as has been demonstrated for SARS-CoV (Channappanavar et al., 2016). Therefore, the enhancement of the innate immune responses that represents the earliest defense line could be a fundamental step to block the progression of immune activation toward more massive and uncontrolled responses that are ultimately harmful to the host organism itself.

Probably only clinical trials with active administration of BCG vaccine will give an evident answer about the protective role of BCG: Giamarellos–Bourboulis *et al.* reported preliminary results about an ongoing clinical trial (ACTIVATE), which evaluates the role of active vaccination with BCG versus placebo on the time to first infection in the elderly. The first data show that the ratio of new infections during the 12-month period of follow-up after BCG vaccination was significantly decreased. The difference in the incidence according to the type of infection showed most of the benefit on the prevention of respiratory infections of probable viral origin. Unfortunately, the outcomes of the ACTIVATE trial did not include the specific assessment for SARS-CoV2 infection (Giamarellos-Bourboulis et al., 2020).

All over the world, several clinical trial protocols (see footnotes 1 and 2) have been developed with active administration of BCG vaccine in selected populations, to evaluate the efficacy during COVID-19. The results of these trials will clarify whether or not there is a real protection by BCG vaccine against SARS-CoV2 infection.

However, in the light of current knowledge and data from our study, we consider appropriate to follow the WHO warning about indiscriminate use of BCG vaccine, until clear evidence of protection by BCG vaccination against COVID-19 is fully demonstrated.

## Data Availability

The raw data supporting the conclusions of this article will be made available by the authors, without undue reservation.

## Footnotes

1 Reducing healthcare workers absenteeism in the COVID-19 pandemic through BCG vaccine (BCG-CORONA). Radboud University, the Netherlands. ClinicalTrials.gov Identifier: NCT04328441.

2 BCG vaccination to protect healthcare workers against COVID-19 (BRACE). Murdoch Children’s Research Institute, Australia. ClinicalTrials.gov Identifier: NCT04327206.
